# Anesthetic management of gigantic pheochromocytoma resection with inferior vena cava and right atrium tumor thrombosis: a case report

**DOI:** 10.1186/s12871-019-0742-6

**Published:** 2019-05-10

**Authors:** Jingli Chen, Caihua Liu, Chang Liu, Quanyuan Fu, Dingwei Pei, Linyun Ren, Hong Yan

**Affiliations:** Department of Anaesthesia, The Central Hospital of Wuhan, Tongji Medical College, Huazhong University of Science and Technology, Wuhan, 430014 Hubei China

**Keywords:** Adrenal tumor, Malignant phaeochromocytoma, Inferior vena cava, Right atrium, Tumor thrombus

## Abstract

**Background:**

This report describes one case of anesthetic management about surgical resection of a malignant phaeochromocytoma with tumor extension into vena cava and right atrium in a patient. Report for anesthetic management is limited in these patients under surgical resection until now.

**Case presentation:**

In September 2015, a 24-year-old male presented to the department of cardiology with right flank pain and hypertensive urgency in our hospital. Contrast-enhanced CT abdomen and MRI abdomen revealed a mass phaeochromocytoma in right adrenal, which invaded the right inferior vena cava(IVC)wall along with IVC thrombus. Echocardiography shown no abnormal detection. Finally, this patient gave up the surgical resection of phaeochromocytoma and chose the expectant treatment. In April 2018, this patient once again presented to the emergence department in our hospital, he had experienced persistent cough and intermittent wheezing for 5 h. Contrast-enhanced CT and echocardiography shown existing IVC thrombus had extended into the right atrium. After the careful preoperative preparation, adrenalectomy with complete thrombus excision by inferior vena cava exploration and right atriotomy were performed successfully by a multidisciplinary team. After one month post-operation care, this patient healthily left our hospital.

**Conclusion:**

To the best of our knowledge, the occurrence of pheochromocytoma with IVC and right atrium thrombosis has not been reported in mainland China so far. This clinical case may supply a rare reference experience for surgical treatment and anesthetic management in the group of phaeochromocytoma patient with distance vascular extension.

## Background

Pheochromocytoma is a rare tumor, which produce catecholamine in the adrenal medulla. Prevalence of the disease may vary but approximately 1 to 2 per 100,000 individuals are diagnosed annually [1]. The classic hallmark of the disease is headache, sudden perspiration, and tachycardia [2]. The disease is commonly diagnosed biochemically by plasma-free metanephrines or 24-h urine fractionated metanephrines and localized by imaging modalities like contrast-enhanced CT, MRI, or metaiodobenzylguanidine scintigraphy [3, 4].

Thrombosis of inferior vena cava (IVC) is common pathological phenomena in clinical diagnosis, which normally has comparable etiological factors to lower limb deep venous thrombosis [5]. The hypercoagulable state of blood or inflammatory processes, neoplastic abnormalities, and vessel injury have all been implicated as primary mechanism in the pathophysiology of IVC thrombosis. Based on recently studies report, intravenous extension is a potential mechanism in the evolution of IVC thrombosis. Actually, it is not well-known that endocrine tumor exhibits the ability to extend into vein [6]. However, few rare studies have reported that pheochromocytoma really infiltrates to IVC in patients [7]. Two major mechanism of intravenous extension of pheochromocytoma have been presented, including propagation within the veins and directly invasion through the vessel wall [8].

At present, pheochromocytoma associated with IVC thrombosis have been reported [9–12]. However, the vascular extension of pheochromocytoma into right atrium through IVC has never been reported in main china. We describe a successful perioperative anesthetic management of a rare case of pheochromocytoma associated with IVC and right atrium thrombosis for tumor excision.

## Case presentation

In September 2015, a 24-year-young man was admitted to our hospital with complaints of headache, sweating, anxiety, dizziness. It was confirmed that he had a history of hypertension and type 2 diabetes by clinical syndromes and examination. He was a smoker with no significant family history. His resting pulse rate was 100 beats/min. The patient’s blood pressure was 163/100 mmHg. Physical examination revealed mild lower abdominal tenderness, both edema of lower extremity and collateral formation over abdomen were unremarkable. Hepatomegaly and abdominis hydrops were also absent in the young man.

**Fig. 1:**
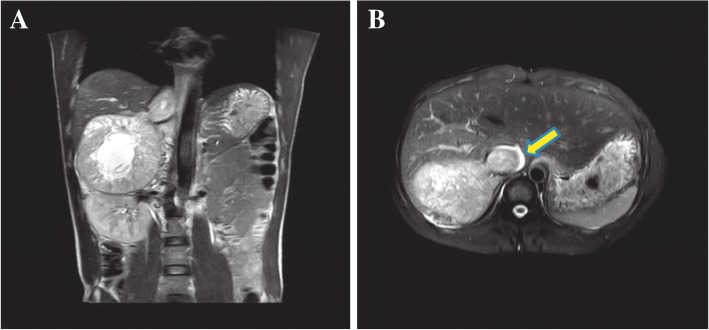
**a**. Magnetic resonance image of the abdomen shows the proximal part of inferior vena cava was widened on longitudinal scan. **b**. Magnetic resonance image of the abdomen indicated tumor thrombus in the inferior vena cava next to the large adrenal phaeochromocytoma on horizontal scan

Abdominal CT revealed a well defined, heterogenous mass lesion of size 11.1 × 10.5 × 11.1 cm at the upper pole of right kidney, which was rooted in right adrenal gland. Vena cava area exhibited filling defect, which indicated the possibility of tumor thrombosis extension from localized tumor. Magnetic resonance imaging (MRI) reveal the proximal part of inferior vena cava was widened (Fig. 1a), and confirmed that intraluminal thrombus is inferior to the right atrium, the distance of extended tumor thrombosis was 6.5 cm approximately (Fig. 1b). Echocardiography indicated this patient had atrial septal defect, the defect size was 0.2 cm, and only showed mild tricuspid regurgitation with 63% EF. Surgical treatment was been suggested. However, the patient gave up the surgical treatment and left hospital.

**Fig. 2:**
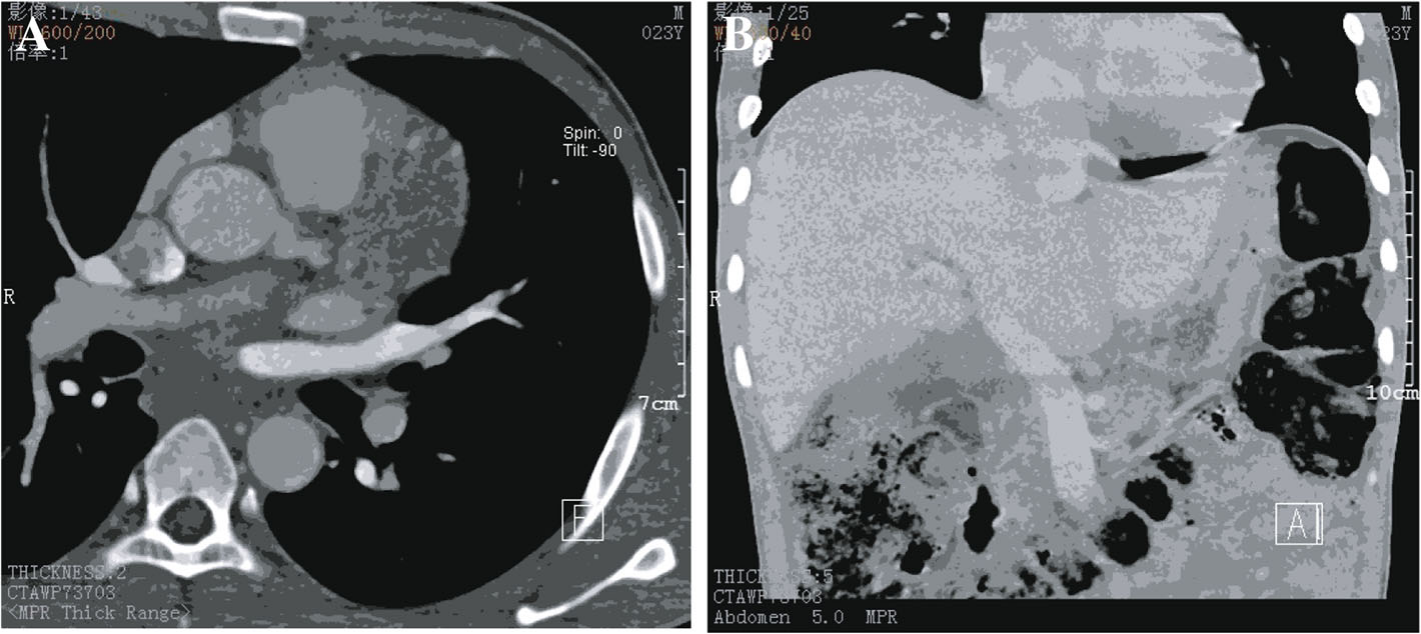
**a**. Contrast-enhanced CT revealed tumor thrombus extended into right atrium on horizontal scan of heart. **b**. Contrast-enhanced CT revealed the distance of tumor thrombus was 13.5 cm on longitudinal scan

**Fig. 3:**
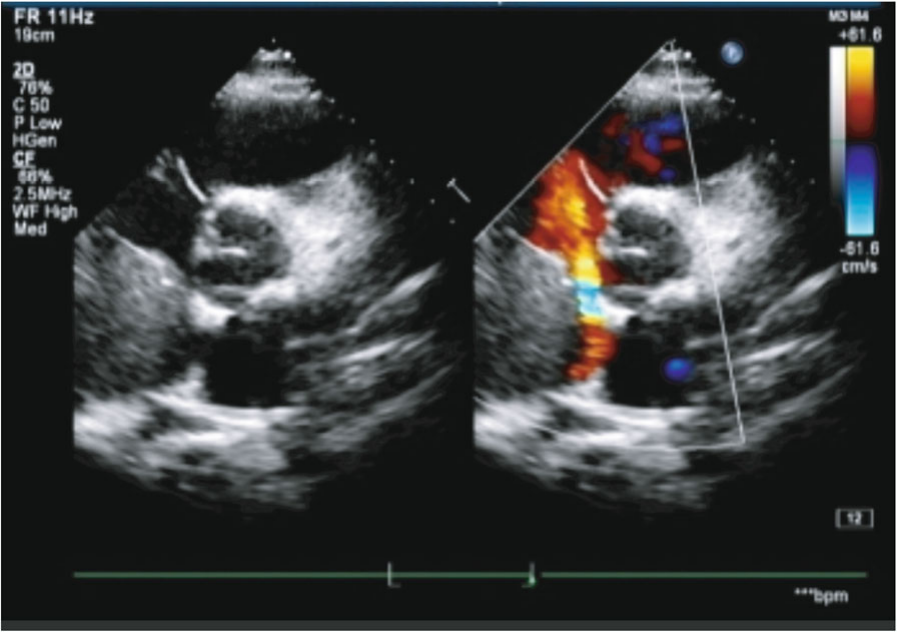
Echocardiography shown existing IVC thrombus had extended into the right atrium

In April 2018, this young man once again presented to the emergence department with persistent cough and intermittent wheezing character for 5 h. At this time, his blood pressure was 160/120 mmHg with a heart rate of 142 beats per minute and SpO2 was 95% on room air. Bilateral lower limb arterial and venous examination was normal. Contrast-enhance CT showed a 12-cm right adrenal mass with tumor thrombosis in IVC, which dubiously extended into right atrium (Fig. 2a), the distance of extended tumor thrombosis was 13.5 cm approximately (Fig. 2b). Echocardiography conformed that existing IVC tumor thrombus had extended into the right atrium (Fig. 3), EF was only 42%. The biochemical evaluation revealed elevated of dopamine 6.03 nmol/L (normal range: 2.09–3.91 nmol/L), adrenaline 15.15 nmol/L (normal range:1.31–2.51 nmol/L), noradrenline 33.17 nmol/L (normal range:1.31–2.30 nmol/L). Preoperative electrocardiography showed supraventricular tachycardia and 24 h Holter monitoring showed no significant cardiac arrhythmia except intermittent junctional rhythm. A primary diagnosis of pheochromocytoma with IVC and right atrium tumor thrombosis was established. The patient finally agreed to receive a surgical treatment. Before receiving the surgical treatment, the patient recurrently presented heart failure in intensive care unit, a combination of alpha-adrenergic and beta-adrenergic blockade and calcium channel blockers were routinely used to maintain the blood pressure and stabilize heart function. In order to avoid the occurrence of hypotension after resecting the phaeochromocytoma, phentolamine was discontinued on the day before the operation. In addition, the beta-adrenergic blockade was continued until the day of operation.

**Fig. 4:**
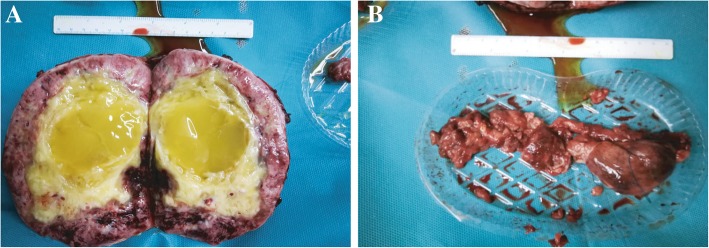
**a**. The image of resected gigantic pheochromocytoma. **b**. The image of removed inferior vena cava and right atrium thrombosis after the operation

Nearly two months later, our anesthetist team finally evaluated the cardiac system of the patient. And we considered that it was an appropriate time to perform the operation based on the relatively favorable physical condition. After being administered intravenous midazolam (1.5 mg), the patient was brought to the operating room. The initial SBP and HR were 144 mmHg and 110 beats/min, respectively, Spo2 was 98% under room air. Two peripheral venous access were established by nurse immediately. After intravenous midazolam (2 mg) and sufentanil (5μg), the left radial artery was cannulated for continuous arterial blood pressure monitoring. Meanwhile, FloTrac system was employed to monitor the cardio output (CO), stroke volume (SV), stroke volume variation (SVV), systemic vascular resistance (SVR). Midazolam (4 mg), sufentanil (70 μg), rocuronium (70 mg) and etomidate (20 mg) were injected via peripheral venous access in sequence. After 2 min of mask positive pressure ventilation, reinforced tracheal tube was intubated. Intravenous infusion of propofol and remifentanil were induced to maintain the anesthesia during the surgery, which were combined with 1–2 MAC of sevoflurane and 100% O_2_. Internal jugular vein puncture was performed to place the central venous catheter and monitor central venous pressure (CVP). Subsequently, transesophageal echocardiography (TEE) probe was inserted to evaluate the ventricular function. Before incising the skin, methylprednisolone (80 mg) and Ulinastatin (7 × 10^5^ unit) were used to resist systematic inflammation reaction. Pantolazole (80 mg) was induced to protect stomach. Subsequently, a midline xiphoid to pubic symphysis incision was induced. Because of the tumor thrombosis in the inferior vena cava and the right atrium, the liver was enlarged and congested secondary to the Budd-Chiari syndrome. A large and well-circumscribed right adrenal phaeochromocytoma was observered clearly, which actually extended through the adrenal vein and lateral wall of inferior vena cava into the right atrium. After the carefully dissociation of tissue, the tumor was successfully dissected from the kidney, liver, and retroperitoneum until it was only attached to the lateral wall of the vena cava at the area of tumor invasion, the resected tumor was shown in Fig. 4a. During the exploration of abdominal, the circulation was extremely unstable. Sodium nitroprusside (0.5-3μg/kg/min) and phentolamine (0.5-3μg/kg/min) were induced to control the SPB in 60-160 mmHg and the DPB in 50–100 mmHg. Nicardipine (1 mg) and phentolamine (1 mg) were also discontinuously intravenous injected to control the blood press cooperatively. Esmolol (50-300μg/kg/min) was induced to revolt tachycardia, but the control of heat rate was unsatisfactory, the heat rate was maintained in 90–140 beats/min. During the resection, the cardiac function and hemodynamic parameters were not relative stable (CO: 3.3–4.5 L/min, SV: 60-82 ml, SVV: 8–26%, SVR: 1023–1368 dynes-s/cm ^5^). Blood gas analysis was performed hourly (PH: 7.34–7.23, PaO_2_:345-222 mmHg, PaCO_2_:37–41 mmHg, SaO_2_:97.9–100% ctHb: 8.1–13.4 g/L, cK^+^: 3.1–5.1 mmol/L, cNa^+^: 135–141 mmol/L, cCa^+^: 0.97–1.23 mmol/L, cLac: 0.8–5.7 mmol/L), these related parameters was modulated to approach the normal range. Subsequently, the cardiac surgeon continued to perform median sternotomy. After systemic heparinisation, the right femoral vein, superior vena cava, and the aorta were cannulated to establish cardiopulmonary bypass. Before the aorta was cross-clamped, the patient was gradually cooled to 32 °C. After the infusion of cold cardioplegia into aortic root, cardiac arrest was achieved. Meanwhile, cardiopulmonary bypass was induced to support the circulation system. By cutting open the right atrium, tumor thrombosis at the right atrium was then resected, the elimination of tumor thrombosis in the vena cava was performed subsequently. The removal tumor thrombosis form IVC and right atrium was shown in Fig. 4b. After repairment of right atrium and vene cava, The patient was weaned off from cardiopulmonary bypass after full re-warming. During the cardiopulmonary bypass, the blood pressure remained relatively stable with any severe hemodynamic disorder. In summarize, the operating time of this patient was totally 11.5 h, the blood loss was approximately 10,000 mL. The diagnosis of phaeochromocytoma was finally confirmed by immunohistochemical method. After one month post-operation care, the patient healthily left our hospital.

## Discussion and conclusion

Pheochromocytoma represents very significant challenges to the anesthetist, which are not uncommon in anesthetic practice. Generally, the laparoscopic approach is preferred for most pheochromocytoma resections [13]. The anesthetic management of the surgical approach has improved remarkably over the years, in conjunction with the evolution of surgical techniques. However, the pheochromocytoma combined with cardiovascular extension was rare. The perioperative anesthetic management will be very hard because of the vascular obstruction induced severe hemodynamic disturbance. In this case, the patient presented gigantic pheochromocytoma with inferior vena cava and right atrium thrombosis. Actually, limited number of case reports on malignant phaeochromocytoma with extension into the right atrium were been reported in the literature. This present report is believed to be the first case of a malignant phaeochromocytoma extending into the right atrium in mainland china.

Surgical resection has been proved to the efficient treatment option for phaeochromocytoma patients [14]. Up to now, various techniques of tumor resection have been applied to limit blood loss and eliminate the tumor completely. However, complexity of anesthetic management is largely depended on surgical approach. In this case, the mass of phaeochromocytomas was nearly 12 cm diameter, and tumor thrombus extended into the right atrium. The radical surgery with the use of cardiopulmonary bypass with hypothermic circulatory arrest is recommended based on previous case report [15]. This surgical approach was highly risky, and anesthetic management was sufficiently challenging.

In the case, the primary anesthesic goal is the delivery of an anesthetic which provides stable hemodynamics in the face of catecholamine surges following tumor handling and opposite scenario following tumor ligation. It is necessary to prepare careful planning with surgical team. Relief from anxiety prior to anesthetic induction is a key component, as apprehension can predispose to catecholamine surges. We made a judicious dose of IV midazolam (1.5 mg) prior to transfer to the operating suite creates a calm patient less prone to hypertensive crises at induction. Traditional vasodilator set up includes nitroglycerin, sodium nitroprusside, nicardipine, diltiazem as indicated, esmolol infusion for heart rate control, magnesium sulfate, and vasoconstrictors such as norepinephrine and vasopressin. For anesthetic induction, limitation of the hemodynamic stresses of direct laryngoscopy should be considered. Propofol and etomidate were recommended. Propofol has been documented to be safe in these patients [16]. Etomidate has the advantage of conferring cardiovascular stability, especially in volume-depleted patients [17]. All agents that cause histamine release should be avoided. During the operation, we also employed TEE to monitor the heart function and confirm the removal of thrombus after surgery. Importantly, intraoperative TEE has the added advantage of real-time monitoring of intravascular volume status, as well as the earlier detection of myocardial wall motion abnormalities aiding the diagnosis of intraoperative myocardial ischemia. Fortunately, the patient did not show significantly myocardial ischemia and heart failure in the presence of unstable circulation during the operation.

In this case, the successful anesthetic management is mainly due to the positive circulation regulation. In generally, tumor manipulation usually generates a far more dramatic pressor response, which is related to increases in plasma levels of norepinephrine and epinephrine [18]. Significant reductions in cardiac output, and associated left ventricular systolic and diastolic dysfunction may be observed in this situation. Acute hemodynamic crises during resection are not uncommon and must be immediately treated. During the process of tumor resection, severe bradycardia accompanied by hypertension and tachyarrhythmias are the common hemodynamic disorder. Deepening the depth of anesthesia and rapidly administering direct arterial vasodilators are the efficient response to control unstable hemodynamics in this condition. Traditionally, sodium nitroprusside is the key drug in conjunction with nitroglycerin, to reduce preload of heart. In addition, the ligation of the tumor bleeding by surgeon and anesthetic-induced persistent vasodilation normally induced the sudden hypotension. The administration of large-volume fluid prior to tumor ligation will be useful to avoid the occurrence of sudden hypotension, as well as stopping the infusion of all vasodilators in due course. In addition, it had been suggested that massive fluid treatment was more effective strategy than vasopressor treatment to reverse the hypotension in these situations.

In conclusion, understanding of the physiology of this type of patients is essential to perform a successful anesthetic management. Meanwhile, we recommend that all this type of patients should be optimized preoperatively and intraoperative precautions taken, including drug intervene and hemodynamic monitoring, which may ensure a stable perioperative course and life safety.

## Abbreviations

CO: Cardio output
CVP: Central venous pressure
IVC: Inferior vena cava
MRI: Magnetic resonance imaging
SV: Stroke volume
SVR: Systemic vascular resistance
SVV: Stroke volume variation
TEE: Transesophageal echocardiography
